# Decision-making performance and motor skill consistency in basketball short pass–shot sequences under different levels of mental fatigue: an integrated psychophysiological dynamics analysis

**DOI:** 10.3389/fpsyg.2026.1831767

**Published:** 2026-06-03

**Authors:** Xinyu Zhou, Shaofei Hou, Yuanbin Sang, Jiahao Jiang, Liao Hu, Lun Li

**Affiliations:** 1 School of Physical Education, China University of Geosciences, Wuhan, China; 2 School of Physical Education, Hubei University of Education, Wuhan, China

**Keywords:** basketball performance, decision-making, heart rate variability, mental fatigue, motor skill consistency, psychophysiological dynamics

## Abstract

**Background:**

Basketball performance depends on the rapid coupling of perceptual decision-making and sequential motor execution. Mental fatigue induced by prolonged cognitive effort may impair both judgment and movement stability, yet its integrated effects on decision-making and motor skill consistency during basketball pass-shot sequences remain insufficiently understood.

**Methods:**

27trained male university basketball players completed low-, moderate-, and high-mental-fatigue conditions in a within-subject crossover design utilizing standardized cognitive tasks. Performance was assessed using a decision-embedded short pass-shot sequence, while synchronized kinematic and psychophysiological data were collected to characterize behavioral output, movement consistency, and autonomic responses.

**Results:**

Increasing mental fatigue significantly reduced decision-making accuracy, prolonged reaction time, decreased sequence success rate, and weakened motor skill consistency. Greater fatigue was also associated with higher temporal variability and larger kinetic fluctuations. At the physiological level, these performance declines corresponded with shifts in autonomic markers—specifically, increased electrodermal activity and decreased heart rate variability—which are suggestive of elevated sympathetic arousal and vagal withdrawal. This pattern points toward a coordinated deterioration across cognitive, motor, and autonomic domains.

**Conclusion:**

Mental fatigue exerts a progressive negative influence on basketball performance, extending from impaired tactical judgment to less stable motor execution and altered autonomic regulation. These findings highlight potential behavioral and psychophysiological pathways linking cognitive exertion to performance decline, offering practical implications for holistic fatigue monitoring in basketball training.

## 1 Introduction

Basketball performance relies heavily on the rapid coupling of perceptual decision-making and sequential motor execution under severe time pressure. While the physical demands of the sport are well documented, the continuous cognitive engagement required to process dynamic spatial information and execute complex skill chains increasingly exposes athletes to mental fatigue. Consequently, understanding how cognitive exertion compromises the integration of tactical choices and motor control has become a critical focus in contemporary sport science. Such continuous cognitive engagement requires sustained attention, inhibition of irrelevant information, and flexible updating of situational representations, which may gradually deplete cognitive resources and contribute to mental fatigue. In competitive settings, where athletes are exposed to both physical strain and cognitive load, understanding how mental fatigue affects performance has become an important issue in sport science research (Yuan et al., 2023; Guo and Wang, 2025; Hemmat et al., 2025; Zhao, 2025).

Mental fatigue is generally described as a psychobiological state caused by prolonged cognitive effort and is commonly associated with increased subjective tiredness, impaired executive function, and reduced attentional control. In basketball, these impairments may be especially consequential because successful performance often depends on rapid and accurate decisions under time pressure. Mentally fatigued players may show poorer working memory, weaker inhibitory control, and slower response selection, which can reduce the accuracy of passing and shooting decisions (Boffet et al., 2025; Vorreuther et al., 2025). Beyond decision-making, mental fatigue may also disrupt the stability of motor execution. From a motor control perspective, fatigue can interfere with the central regulation of coordinated movement, thereby disturbing the automation and precision of skilled actions. In sequential tasks such as short-pass and shot execution, impaired decisions and unstable movement patterns may co-occur, creating a cascading decline in overall performance (Zhang et al., 2025).

Although previous studies have demonstrated adverse effects of mental fatigue on sport performance, three important limitations remain in basketball research. First, many studies rely on fragmented task paradigms that examine isolated skills, such as shooting or dribbling, rather than decision-embedded action sequences that better reflect real-game demands. Second, outcome assessment has often focused on final performance indices, especially shooting accuracy, while giving limited attention to motor skill consistency and kinematic variability, which may be more sensitive indicators of fatigue-related disruption (Pan et al., 2025). Third, the psychophysiological mechanisms underlying fatigue-related performance decline remain insufficiently integrated. Existing studies have often described either psychological changes or physiological responses, but rarely examined how subjective fatigue, autonomic regulation, and behavioral impairment are dynamically linked within the same framework. A more integrated psychophysiological approach is therefore needed to clarify how mental fatigue propagates from cognitive processing to motor execution.

The present study addressed these gaps by examining the effects of different levels of mental fatigue on decision-making performance and motor skill consistency during basketball short pass-shot sequences using a combined psychophysiological design (Bian et al., 2025). We hypothesized that escalating levels of cognitive fatigue would progressively impair tactical decision-making, evidenced by reduced choice accuracy and prolonged response latency. Furthermore, we postulated that this cognitive depletion would disrupt motor skill consistency, manifesting as increased kinematic variability and lower overall sequence success rates. Finally, we expected these behavioral decrements to correspond directly with objective autonomic dysregulation, specifically reflected by altered heart rate variability and electrodermal activity across the fatiguing conditions. In addition, individual differences in fatigue susceptibility were explored to identify potential variability in performance resilience under mentally fatiguing conditions.

## 2 Methodology

### 2.1 Study design

A within-subject randomized crossover design was used to investigate the effects of low, moderate, and high mental fatigue on basketball decision-making, motor skill consistency, and psychophysiological responses during short-pass–shot sequences. Each participant completed three visits, with one fatigue condition tested per visit. The condition order was randomized using a counterbalanced design, and visits were separated by at least 7 days to minimize residual fatigue and learning effects. To eliminate potential order effects, participants were randomly assigned to one of three experimental sequences: Sequence A (Low–Moderate–High mental fatigue), Sequence B (Moderate–High–Low), or Sequence C (High–Low–Moderate), ensuring that each fatigue level appeared in each visit position an equal number of times across the cohort. In each visit, participants underwent baseline assessment, equipment calibration, mental fatigue induction and manipulation check, standardized warm-up, task performance, and recovery recording. This design enabled within-subject comparison across fatigue conditions while controlling for individual differences.

### 2.2 Participants

A total of 27 young male basketball players with regular training experience were recruited from a university in Hubei Province, China. The sample size was determined *a priori* using G^*^Power software (version 3.1.9.7). Based on a repeated-measures within-subject design, assuming a moderate effect size (f = 0.25), an alpha level of 0.05, and a statistical power of 0.80, a minimum of 24 participants was required to detect significant main effects across the three fatigue conditions. To account for potential dropouts or data acquisition failures, 30 participants were initially recruited. Ultimately, 27 participants successfully completed all experimental visits with valid synchronized data, thereby providing adequate statistical power to test the primary hypotheses. All participants were able to independently complete the standardized short-pass–receive–shot sequence. The inclusion criteria were rigorously operationalized as follows: (a) a minimum of 3 years of structured basketball training, alongside active current participation in university-level or regional amateur basketball leagues; (b) objectively demonstrated technical proficiency, defined as achieving a minimum baseline accuracy of 80% in standard short-passing and 40% in unguarded spot-up shooting during a standardized 20-trial pre-screening assessment; and (c) no history of neurological, cardiovascular, endocrine, or recent musculoskeletal disorders that could affect exercise performance. Exclusion criteria included acute pain or injury affecting training within the previous 2 weeks, use of medications influencing attention or executive function, severe sleep deprivation (defined as total sleep time < 6 h on the night preceding the experiment) or recent circadian disruption, and failure to complete the protocol or meet signal-quality requirements during pilot testing. The study protocol was approved by the Ethics Committee of China University of Geosciences (Approval No: CUG-TY-2026-01), and written informed consent was obtained from all participants.

Before the first visit, demographic characteristics and training background were recorded, including age, sex, height, weight, body mass index, dominant hand, playing position, years of training, and weekly training frequency. To minimize external influences on mental fatigue manipulation and task performance, participants were instructed to avoid strenuous exercise before each visit, limit caffeine intake, and report their sleep duration on the previous night. Baseline autonomic and psychological measures were also collected, including resting heart rate, heart rate variability, and sleep duration. Habitual sleep quality was assessed using the Pittsburgh Sleep Quality Index (PSQI), a validated 19-item self-report instrument (Mollayeva et al., 2016). Furthermore, current subjective fatigue and perceived stress were assessed using a 10-cm visual analog scale (VAS) anchored by ‘not at all' (0) and ‘extremely' (10). Participants completed these scales under investigator supervision after standardized instructions to ensure data consistency. All participants were familiarized with the scale procedures before formal testing. Baseline skill performance was quantified using short-pass accuracy, set-shot percentage, and pass–shot sequence success rate. Task outcomes were recorded independently by two trained assessors according to predefined scoring criteria, and discrepancies were resolved by review of the video record to reduce counting error.

To control for order effects, the study used a within-subject randomized crossover design with balanced allocation of condition sequence. No significant between-sequence differences were observed in demographic characteristics, training background, baseline psychological state, physiological measures, or baseline skill performance (Table 1), indicating good comparability before exposure to the different mental fatigue conditions.

**Table 1:** Participant characteristics and baseline equivalence.

Characteristic	Total (*N* = 27)	Sequence A (*n* = 9)	Sequence B (*n* = 9)	Sequence C (*n* = 9)	*P* value^*^
Age (years)	21.6 ± 2.3	21.4 ± 2.1	21.9 ± 2.5	21.6 ± 2.4	0.86
Sex, male, *n* (%)	27 (100.0)	9 (100.0)	9 (100.0)	9 (100.0)	–
Height (cm)	177.8 ± 6.4	176.9 ± 6.8	178.6 ± 6.2	177.8 ± 6.5	0.78
Body mass (kg)	71.9 ± 8.1	70.6 ± 7.5	73.1 ± 8.9	72.0 ± 8.2	0.74
BMI (kg/m^2^)	22.7 ± 2.0	22.5 ± 1.8	22.9 ± 2.3	22.7 ± 2.0	0.88
Dominant hand, right, *n* (%)	24 (88.9)	8 (88.9)	8 (88.9)	8 (88.9)	1
Playing position, *n* (%)					0.97
+ Guard	12 (44.4)	4 (44.4)	4 (44.4)	4 (44.4)	
+ Forward	10 (37.0)	3 (33.3)	4 (44.4)	3 (33.3)	
+ Center	5 (18.5)	2 (22.2)	1 (11.1)	2 (22.2)	
Basketball training experience (years)	6.8 ± 2.4	6.6 ± 2.1	7.1 ± 2.6	6.7 ± 2.5	0.84
Weekly training frequency (sessions/week)	3.6 ± 1.2	3.4 ± 1.1	3.7 ± 1.3	3.6 ± 1.2	0.83
Weekly training volume (h/week)	7.8 ± 2.6	7.5 ± 2.4	8.1 ± 2.8	7.7 ± 2.7	0.79
Sleep duration (h/night, last 7 days)	7.1 ± 0.8	7.0 ± 0.7	7.2 ± 0.9	7.1 ± 0.8	0.68
Pittsburgh sleep quality index (PSQI)	4.6 ± 1.8	4.7 ± 1.9	4.4 ± 1.7	4.6 ± 1.8	0.92
Caffeine intake (cups/day)	0.9 ± 0.7	0.8 ± 0.6	1.0 ± 0.8	0.9 ± 0.7	0.73
Resting heart rate (bpm)	62.4 ± 7.9	63.1 ± 8.2	61.7 ± 7.4	62.3 ± 8.1	0.9
Baseline HRV (RMSSD, ms)	44.9 ± 16.1	46.3 ± 17.4	43.1 ± 14.8	45.2 ± 16.3	0.88
Baseline VAS fatigue (0–10)	1.7 ± 1.0	1.6 ± 0.9	1.8 ± 1.1	1.7 ± 1.0	0.89
Baseline perceived stress (0–10)	2.3 ± 1.3	2.2 ± 1.2	2.4 ± 1.4	2.3 ± 1.3	0.91
Baseline short pass accuracy (%)	86.9 ± 6.7	86.3 ± 6.1	87.6 ± 7.4	86.9 ± 6.9	0.87
Baseline spot-up shot percentage (%)	53.8 ± 8.4	54.1 ± 8.0	53.2 ± 8.9	54.0 ± 8.6	0.95
Baseline pass–shot sequence success (%)	48.6 ± 7.9	49.2 ± 7.3	47.8 ± 8.5	48.8 ± 8.0	0.9
Baseline decision accuracy (%)	81.2 ± 7.1	80.6 ± 6.8	82.1 ± 7.6	80.8 ± 7.0	0.82
Baseline decision latency (ms)	684 ± 92	693 ± 88	672 ± 95	686 ± 94	0.84

### 2.3 Mental fatigue manipulation and manipulation check

Mental fatigue was induced through a computer-based version of the Stroop Color-Word Task, a well-established cognitive challenge known to deplete executive resources in sports-specific contexts. To establish a verifiable gradient of mental exhaustion across the crossover design, three experimental conditions were implemented, with participants exposed to a single, distinct condition per visit: (a) Low Mental Fatigue (Low MF), which served as a control involving 60 minutes of watching a neutral documentary (e.g., 'Nature's Great Events') to maintain a state of low cognitive demand; (b) Moderate Mental Fatigue (Moderate MF), consisting of 30 min of the continuous Stroop task; and (c) High Mental Fatigue (High MF), requiring 90 min of sustained performance on the same task. The task difficulty was maintained by requiring participants to respond to the ink color of words while ignoring their semantic meaning, a process that necessitates high levels of inhibitory control and sustained attention. This duration-based approach follows the protocol established by Marcora et al., ensuring that the cognitive effort was sufficient to induce measurable psychobiological fatigue without causing physical exhaustion. All experimental procedures were conducted in a standardized laboratory environment with controlled lighting, consistent temporal pacing, and uniform investigator feedback to minimize extraneous variability.

To achieve efficient induction and clear intensity stratification, this study used a dual-channel manipulation strategy that included both subjective and behavioral evaluation. Subjective psychological state was quantified by measuring perceived fatigue and task load pre- and post-induction. This assessment captured multidimensional exertion, encompassing physical tiredness, attentional focus, and cognitive effort, thereby accurately tracking the progressive onset of mental fatigue. Behavioral metrics from the Stroop task, including mean reaction time (RT), accuracy, and the rate of inhibitory errors, were recorded to objectively verify cognitive performance decline. These behavioral indices, combined with pre- and post-induction subjective fatigue ratings, provided a multi-dimensional confirmation of the fatigue state. A successful manipulation was defined by a significant increase in subjective exhaustion and a concomitant decrease in cognitive task efficiency across the three conditions, confirming the existence of a stable fatigue gradient prior to the motor task.

To ensure the reproducibility of the fatigue induction and minimize the interference of extraneous psychological variables, several rigorous experimental controls were enforced. Beyond the baseline standardization of sleep and caffeine intake, participants strictly adhered to a regulated laboratory protocol during each visit. Specifically, a controlled rest period was implemented between the fatigue induction phase and the commencement of the basketball sequence. This interval was designed to stabilize the participant's physiological state while preventing any additional cognitive load that might contaminate the manipulated fatigue effect. By maintaining a quiet, standardized environment with minimal investigator interaction, we ensured that the observed performance decrements were primarily attributable to the graded cognitive exhaustion protocol.

### 2.4 Data acquisition and integrated processing workflow

**Figure 1:**
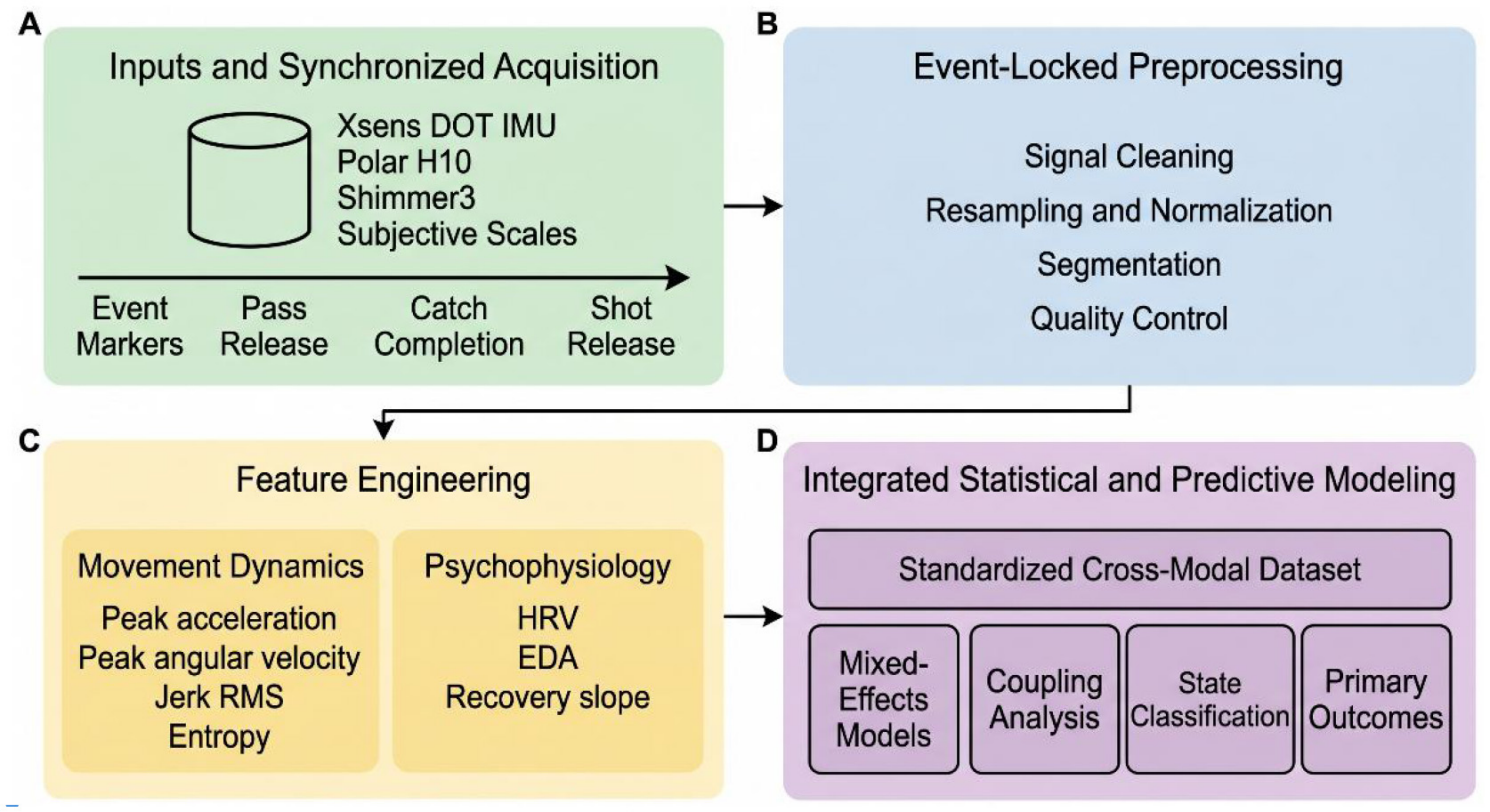
Integrated data acquisition and analytical pipeline for the basketball short pass-shot sequence. The workflow strictly outlines the synchronized raw signal inputs, event-locked preprocessing, feature extraction, and the final inferential and predictive modeling stages employed in the study. **(A)** Inputs and Synchronized Acquisition. **(B)** Event-Locked Preprocessing. **(C)** Feature Engineering. **(D)** Integrated Statistical and Predictive Modeling.

Data acquisition was strictly event-locked. The temporal alignment of these discrete task events across multiple measurement modalities provided the necessary synchronization to adequately model both decision-making performance and motor skill consistency. Sensor arrangement and coordinate system definition are shown in Figure 1. Kinematic data were captured using high-precision 9-axis inertial measurement units (Xsens DOT, Xsens Technologies B.V., Enschede, Netherlands), which comprise a tri-axial accelerometer, gyroscope, and magnetometer. These sensors were synchronized and sampled at 100 Hz, with a full-scale range of ± 16 g and ± 2000 /s, respectively. These devices have been previously validated for high-velocity sports movements and functional tasks, demonstrating excellent intra-instrument reliability and concurrent validity against optical motion capture systems (Ribeiro-Fernandes et al., 2022). The wrist sensor was secured to the dorsal aspect of the dominant forearm, while a second trunk-mounted IMU was positioned over the third thoracic vertebra to monitor postural stability. To guarantee uniform data acquisition across the cohort, the sensor array was strictly standardized for all experimental trials. This required every participant to simultaneously wear the wrist IMU, the trunk IMU, the cardiac chest strap, and the palmar electrodermal sensors. No devices were deployed as optional components, ensuring that the kinematic and autonomic responses were comprehensively recorded for every phase of the motor sequence. Cardiac activity was monitored using a Polar H10 heart rate strap (Polar Electro Oy, Kempele, Finland), widely recognized as the gold standard for non-invasive R-R interval detection with proven signal quality and criterion validity during high-intensity exercise (Gilgen-Ammann et al., 2019). Electrodermal activity (EDA) was recorded via a Shimmer3 GSR+ unit (Shimmer Research, Dublin, Ireland) using 8mm Ag/AgCl electrodes placed on the non-dominant palm, an ambulatory measurement system validated for the continuous assessment of sympathetic arousal (Ronca et al., 2023). HRV analysis was performed using Kubios HRV Premium software (Version 3.5, Kuopio, Finland), a scientifically validated tool for cardiac autonomic parameter extraction, where R-R intervals were preprocessed with a validated threshold-based artifact correction algorithm (Lipponen and Tarvainen, 2019). Time-domain (RMSSD) and frequency-domain (LF/HF ratio) indices were derived according to the Task Force guidelines. EDA signals were decomposed into tonic and phasic components using Continuous Decomposition Analysis (CDA) in the Ledalab toolbox, a well-established algorithm validated for separating superimposed skin conductance responses (Benedek and Kaernbach, 2010), ensuring that the phasic skin conductance response (SCR) rate accurately reflected cognitive-load-induced arousal. To ensure the comparability of signal data under different visit conditions and various states of psychological fatigue, all devices were subjected to standard wearing calibration and signal quality inspection at the beginning of each visit, and the wearing position, fixation method, and sampling settings remained consistent across all visits.

The experimental task required participants to respond to dynamic visual stimuli projected onto a 3 × 3 meter LED screen located 5 meters ahead. Each trial began with a random directional cue (left/right) indicating the pass receiver, followed by a defensive closing-out simulation that forced a tactical decision between a quick shot or a return pass. A total of 24 trials were performed per fatigue condition, organized into three blocks of eight to prevent immediate physical exhaustion. Success was strictly defined as the completion of both an accurate chest pass (hitting the target center) and a successful field goal within a total temporal window of 3.0 s. To carry out more in-depth analyses, we added event markers at several crucial moments during each trial of the experiment this time. Kinematic and psychophysiological signals were subsequently segmented and aligned relative to these specific event markers. Specifically, discrete event-locked data windows were extracted spanning from 0.3 s prior to 0.8 s after each critical marker—pass release, catch completion, and shot release. This precise temporal alignment facilitated the direct evaluation of motor control strategies and execution stability across the varying levels of mental fatigue.

**Figure 2:**
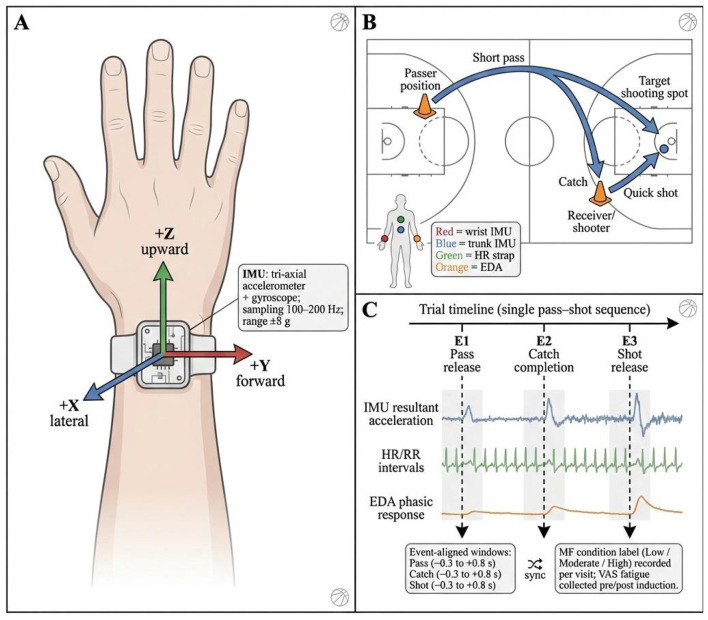
Task-specific sensor deployment and event timing alignment for the short-pass-shot sequence under mental fatigue. **(A)** Sensor placement and coordinate system definition for the wrist-worn IMU sensor. **(B)** Basketball court diagram illustrating the pass and shot scenario with sensor placements on the human figure. **(C)** Trial timeline and event-locked data windows for the pass, catch, and shot segments.

As shown in Figure 2, the data processing pipeline followed a strictly defined sequence encompassing preprocessing, segment alignment, feature engineering, and final model fusion. Initial signal preprocessing involved systematic denoising and artifact rejection. This pipeline included bandpass filtering, the identification and removal of movement artifacts, missing data imputation, and routine resampling. Normalization procedures were subsequently applied to mitigate the confounding effects of sensor drift and inter-subject baseline variability prior to feature extraction. Following artifact rejection, the continuous signals were segmented into discrete time windows locked to the pass, receive, and shot event markers. Trial-level quality control was subsequently performed to exclude any corrupted segments, ensuring that only high-fidelity data were retained for cross-condition statistical comparisons.

Feature engineering was conducted simultaneously across kinematic and psychophysiological domains. Metrics were calculated independently for each movement phase before being aggregated at the trial level. Kinematic features were extracted across the temporal and spatial domains to quantify the magnitude, rhythm, and consistency of the multi-joint movements. Concurrently, psychophysiological features were computed to assess autonomic nervous system regulation, providing objective indices of sympathetic arousal and parasympathetic withdrawal under varying levels of cognitive load. Following feature extraction, cross-modal variables were standardized and fused to construct an integrated analytical dataset. For statistical inference, within-subject mixed-effects models were fitted to estimate the primary effects of mental fatigue on both decision-making and skill consistency, adjusting for cross-trial-block interactions. Furthermore, cross-domain coupling analyses were conducted to evaluate the correlated degradation across the psychological, physiological, and motor performance domains. To supplement these inferential statistics, ensemble machine learning classifiers—specifically Random Forest and Gradient Boosting algorithms—were trained to discriminate fatigue-induced performance states. These classification models served as a robust auxiliary validation step to confirm the predictive utility of the extracted psychophysiological features without replacing the primary mixed-effects analysis.

### 2.5 Outcome definitions

The outcome indicators of this study were divided into four dimensions according to the central objective of the short-pass receiving and passing skill chain: decision-making performance, execution results of skills, consistency of skill application, and psychological-physiological state. Evaluations of the kind were made in different states of psychological fatigue.

Decision-making metrics served as primary outcome variables. Decision accuracy was defined as the proportion of trials in which a participant selected the tactically optimal response to the presented situational stimulus. Furthermore, decision latency was operationalized as the temporal interval between the onset of the visual stimulus and the initiation of the participant's motor response. To comprehensively evaluate the speed-accuracy trade-off, a decision efficiency index was subsequently derived by dividing the decision accuracy by the corresponding decision latency for each experimental condition. Choice quality score was quantified using a weighted 10-point scale where three expert coaches (FIBA Level 1 or higher) independently rated the tactical appropriateness of each decision based on the simulated defender's proximity; the final score represented the mean of these ratings. High-risk choice rate was defined as the percentage of trials where participants opted for a shot execution despite the defender being within a high-pressure radius (0.5–1.0m), a choice typically associated with a lower probability of success under mental strain. Cross-condition comparisons can then be conducted under the same evaluation criteria.

Skill execution outcomes were operationalized through precise performance metrics for each phase of the task. Pass accuracy was determined by the spatial landing error relative to the target center, while catch error rate was defined as the proportion of trials involving a fumble or failed reception. Shooting performance was recorded as the binary field goal percentage. Finally, the overall sequence success rate was calculated as the percentage of trials where the participant seamlessly executed an accurate pass, a clean catch, and a successful shot within the strict 3.0-s time limit.

To evaluate the disruption of overlearned motor sequences under cognitive exhaustion, we analyzed temporal and spatial variability across the execution phases. Phase timing variability was calculated to quantify temporal deviations between the passing, catching, and shooting segments, thereby reflecting the stability of movement rhythm. Kinematic consistency was quantitatively assessed using the coefficient of variation and standard deviation of a predefined set of biomechanical markers. Specifically, peak acceleration, peak angular velocity, jerk root-mean-square, and entropy-based stability indices were extracted, given their established mechanical relevance to shooting success. To account for baseline inter-subject variability, these consistency metrics were calculated independently for the passing, catching, and shooting phases within each participant before being aggregated into a composite trial-level score.

Psychophysiological indicators were employed to continuously monitor autonomic nervous system regulation and arousal dynamics throughout the protocol. Specifically, heart rate and heart rate variability served as established indices of cardiac autonomic control, whereas skin conductance was measured to reflect sympathetic arousal. Furthermore, subjective ratings of fatigue and perceived task load were recorded pre- and post-induction to serve as complementary explanatory variables, thereby controlling for baseline inter-subject variability in fatigue susceptibility. All indicators were extracted during the set time windows and aligned with the trials for coupling analysis.

### 2.6 Statistical analysis

All statistical analyses were performed using IBM SPSS Statistics for Windows (Version 27.0; IBM Corp., Armonk, NY, United States) and R software (Version 4.3.2; R Foundation for Statistical Computing, Vienna, Austria). A two-tailed *p* < 0.05 was considered statistically significant. Continuous variables are presented as mean ± standard deviation or median (interquartile range), and categorical variables as frequencies and percentages. To rigorously account for the repeated-measures structure and trial-level nested data, linear mixed-effects models (LMMs) were constructed for continuous outcomes, while generalized linear mixed-effects models (GLMMs) with a logit link function were applied to binomial performance outcomes. Prior to modeling, the normality and homoscedasticity of residuals were assessed using Shapiro-Wilk tests and visual inspection of Q-Q plots; skewed continuous variables were log-transformed where appropriate. In all models, mental fatigue level was designated as the primary fixed effect. To control for potential confounding, visit order and trial block were included as fixed covariates, whereas participant identity was modeled as a random intercept to resolve the non-independence of intra-subject observations. Furthermore, to mitigate Type I error inflation arising from multiple outcome testing, the False Discovery Rate (FDR) procedure was strictly applied to adjust *p*-values across families of related hypotheses. Effect sizes and 95% confidence intervals were generated for all significant fixed effects.

For the multivariable predictive analyses, a purposeful selection strategy was employed to avoid model oversaturation. Candidate psychophysiological predictors were initially screened using bivariate correlations. Only variables demonstrating statistically significant associations with the respective task outcome were advanced to the final adjusted mixed-effects models.

Finally, exploratory machine learning classification was conducted to map the multidimensional psychophysiological feature space onto categorical performance states. Given the relatively small sample size, these analyses were executed at the trial-block level rather than the participant level to yield a sufficient number of instances for stable model training. The classification target for decision quality was binarized into poor vs. good states based on a median split of the composite choice score. Similarly, skill-consistency degradation was defined using a pre-established threshold (*z* < −0.5) on the composite consistency index. Input features comprised the z-standardized kinematic and autonomic variables. Random Forest and Gradient Boosting algorithms were implemented using the scikit-learn framework. To rigorously evaluate model generalizability and prevent data leakage, a Leave-One-Subject-Out Cross-Validation (LOSOCV) strategy was utilized. This approach ensured that trials from the same participant never appeared simultaneously in both the training and testing sets, thereby verifying the predictive robustness of the selected features across unseen individuals. Classification performance was evaluated using standard metrics, including precision, recall, and F1-score.

## 3 Results

### 3.1 Effectiveness of mental fatigue manipulation

Manipulation checks were conducted using subjective fatigue, perceived task load, and cognitive task performance to verify the effectiveness of mental fatigue induction. The results showed a clear and stable intensity gradient across conditions, indicating that the manipulation successfully produced different levels of mental fatigue within the same participants.

Subjective ratings showed comparable baseline fatigue across the three conditions before induction. Following the induction protocol, subjective fatigue scores increased in a stepwise manner. *Post-hoc* pairwise comparisons revealed significant differences across all sequential levels. Specifically, the highest fatigue values were observed in the high-fatigue condition, which was significantly greater than both the moderate-fatigue (*p* < 0.01) and low-fatigue (*p* < 0.001) states, while the moderate condition also significantly exceeded the low-fatigue baseline (*p* < 0.01). A similar pattern was observed for pre–post changes in fatigue. Perceived task load also increased with fatigue intensity, including mental demand, effort, frustration, and overall workload, suggesting that higher-intensity induction imposed greater cognitive burden (Table 2).

**Table 2:** Manipulation check: cognitive task performance and subjective mental fatigue under different conditions.

Measure	Low MF (control)	Moderate MF	High MF	*P* value†
Subjective fatigue (VAS, 0–10)				
Pre-induction	1.6 ± 0.9	1.7 ± 1.0	1.6 ± 1.0	0.78
Post-induction	2.4 ± 1.2	4.5 ± 1.4	6.7 ± 1.5	< 0.001
Δ (Post–Pre)	0.8 ± 0.7	2.8 ± 1.0	5.1 ± 1.2	< 0.001
Task load (NASA-TLX, 0–100)				
Mental demand	33.6 ± 9.8	55.2 ± 11.6	71.4 ± 10.9	< 0.001
Effort	38.9 ± 10.7	60.8 ± 12.2	77.3 ± 11.8	< 0.001
Frustration	21.8 ± 9.4	34.7 ± 11.1	48.6 ± 12.7	< 0.001
Overall TLX (mean of subscales)	32.9 ± 8.9	51.6 ± 10.4	66.9 ± 10.8	< 0.001
Cognitive task performance during induction				
Accuracy (%)	95.8 ± 2.6	92.1 ± 3.8	87.3 ± 5.2	< 0.001
Reaction time (ms)	468 ± 54	512 ± 63	583 ± 77	< 0.001
Error rate (%)	4.2 ± 2.6	7.9 ± 3.8	12.7 ± 5.2	< 0.001
Lapse rate (%)‡	2.1 ± 1.9	4.6 ± 3.1	8.3 ± 4.7	< 0.001
Perceived concentration (0–10)				
Post-induction	7.6 ± 1.1	6.3 ± 1.3	5.1 ± 1.4	< 0.001

Behavioral results further corroborated the efficacy of the manipulation. *Post-hoc* analyses confirmed that participants exhibited progressively longer reaction times and higher error rates as fatigue intensified, with significant differences detected not only between the high and low conditions (*p* < 0.001) but also between the moderate and low conditions (*p* < 0.05), thereby validating the establishment of a distinct cognitive impairment gradient. Attention-related errors were particularly pronounced in the high-fatigue condition, indicating reduced attentional stability and information-processing efficiency under sustained cognitive load. Together, these findings confirm that the induction protocol successfully elicited graded mental fatigue at both the subjective and behavioral levels.

### 3.2 Effects of mental fatigue on decision-making and task performance

As shown in Table 3, mental fatigue had a clear adverse effect on decision-making performance during the pass–shot sequence. With increasing fatigue level, participants showed lower decision accuracy and longer decision latency, indicating that mental fatigue impaired both the speed and quality of response selection. The decline was most pronounced under the high-fatigue condition, whereas performance in the low-fatigue condition remained relatively stable. These findings suggest that sustained cognitive load reduced the efficiency of perceptual processing and action selection during sequential basketball performance.

**Table 3:** Decision-making performance in pass–shot sequences across mental fatigue levels.

Decision-making metric	Low MF	Moderate MF	High MF	*P* value
Decision accuracy (%)	82.6 ± 6.8	76.9 ± 7.5	69.8 ± 8.6	< 0.001
Decision latency (ms)	679 ± 88	736 ± 96	812 ± 108	< 0.001
Choice quality score (0–10)	7.6 ± 1.0	6.9 ± 1.1	6.1 ± 1.2	< 0.001
High-risk choice rate (%)	18.4 ± 7.2	22.9 ± 8.1	29.7 ± 9.6	0.002
Late decision rate (%)	9.8 ± 5.4	13.7 ± 6.2	19.6 ± 7.5	< 0.001
Rule-violation errors (*n* per 24 trials)	0.9 ± 0.8	1.4 ± 1.0	2.3 ± 1.3	< 0.001
Correct decision with successful execution (%)	45.7 ± 8.3	39.8 ± 8.7	33.1 ± 9.4	< 0.001
Decision efficiency index (accuracy/latency)	0.123 ± 0.018	0.106 ± 0.019	0.087 ± 0.020	< 0.001
Decision consistency (within-subject SD of latency, ms)	119 ± 36	132 ± 41	151 ± 48	0.006

**Figure 3:**
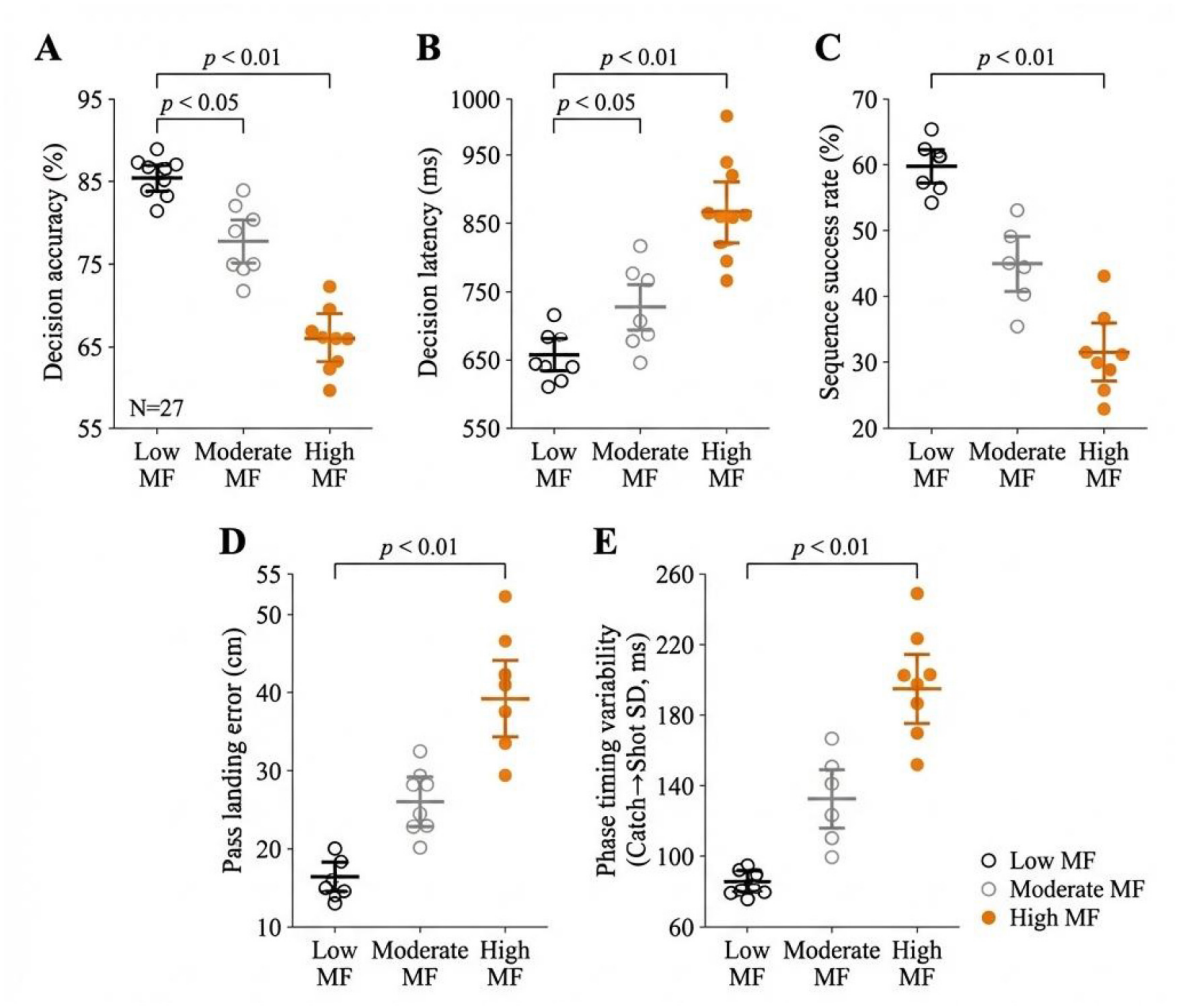
Fatigue-induced alterations in decision-making and skill execution. **(A–E)** depict condition-wise comparisons for decision accuracy, decision latency, sequence success rate, pass landing error, and catch-to-shot timing variability, respectively. Data are presented as individual trial-aggregated data points with superimposed mean ± SD. Brackets indicate exact *post-hoc* significance levels for between-condition differences.

This pattern was further illustrated in Figure 3, which summarizes fatigue-related changes in decision performance and task execution outcomes across trials. Figure 3A shows a progressive decline in decision accuracy as fatigue increased. Figure 3B indicates a corresponding prolongation of decision time, suggesting slower response processing under greater mental fatigue. At the behavioral outcome level, Figure 3C shows a reduction in pass–shot sequence success, and Figure 3D suggests poorer execution quality under fatigued conditions. Overall, the figure demonstrates that mental fatigue affected not only cognitive decision-making but also the successful completion of the integrated basketball sequence.

Together, the results indicate that mental fatigue disrupted both judgment and task execution, supporting the view that decision-making impairment is an important pathway through which fatigue degrades basketball performance.

### 3.3 Effects of mental fatigue on motor skill consistency

As detailed in the consolidated Table 4, escalating mental fatigue systematically degraded both the macroscopic execution outcomes and the microscopic kinematic consistency of the pass–catch–shot sequence. Participants exhibited a progressive decline in overall sequence success rates alongside a concomitant rise in phase-specific execution errors. This behavioral deterioration was fundamentally underpinned by increased motor variability, evidenced by elevated phase timing fluctuations, higher jerk root-mean-square, and diminished entropy-based stability indices under the high-fatigue condition. Collectively, these findings demonstrate that cognitive exhaustion inherently disrupts the stable spatiotemporal organization of sequential movements, leading to compounded mechanical inefficiencies.

**Table 4:** Motor skill execution outcomes and kinematic consistency metrics across mental fatigue levels.

Category/metric	Low MF	Moderate MF	High MF	*P* value
Overall Sequence Performance				
Sequence success rate (%)	48.9 ± 8.3	43.7 ± 8.6	36.5 ± 9.4	< 0.001
Sequence failure due to pass (%)	31.4 ± 9.8	34.9 ± 10.4	38.7 ± 11.1	0.041
Sequence failure due to catch (%)	24.6 ± 8.7	27.9 ± 9.1	32.8 ± 10.2	0.018
Sequence failure due to shot (%)	44.0 ± 10.5	37.2 ± 10.1	28.5 ± 9.7	0.003
Phase-Specific execution outcomes				
Pass accuracy (%)	87.8 ± 6.2	84.1 ± 6.9	79.3 ± 7.6	< 0.001
Pass landing error (cm)	22.6 ± 7.9	26.8 ± 8.6	33.4 ± 10.2	< 0.001
Catch error rate (%)	6.9 ± 4.1	9.8 ± 5.2	14.6 ± 6.8	< 0.001
Time catch → shot (ms)	842 ± 132	903 ± 145	992 ± 167	< 0.001
Shot percentage (%)	54.7 ± 8.6	51.2 ± 8.9	46.1 ± 9.7	0.002
Temporal variability				
Phase timing variability: Pass → Catch (SD, ms)	92.4 ± 24.7	105.8 ± 28.9	128.6 ± 35.4	< 0.001
Phase timing variability: Catch → Shot (SD, ms)	114.7 ± 31.2	129.6 ± 34.8	156.9 ± 41.1	< 0.001
Shot release time variability (SD, ms)	118 ± 39	132 ± 44	154 ± 52	0.010
Kinematic variability & stability				
Peak accel variability (CV, %)	9.6 ± 2.9	11.2 ± 3.2	13.9 ± 3.9	0.001
Peak angular velocity variability (CV, %)	10.8 ± 3.1	12.4 ± 3.5	15.1 ± 4.1	0.002
Jerk RMS variability (CV, %)	12.9 ± 3.6	14.8 ± 4.0	18.0 ± 4.6	< 0.001
Movement smoothness (SPARC, a.u.)	0.58 ± 0.07	0.54 ± 0.08	0.49 ± 0.09	0.004
Sample entropy of accel magnitude (a.u.)	1.21 ± 0.19	1.33 ± 0.21	1.48 ± 0.24	< 0.001
Approximate entropy (a.u.)	0.86 ± 0.14	0.93 ± 0.15	1.05 ± 0.17	< 0.001
Phase stability index (0–1)	0.74 ± 0.09	0.69 ± 0.10	0.62 ± 0.11	0.002
Composite consistency score (*z*, higher = better)	0.42 ± 0.56	0.06 ± 0.61	−0.48 ± 0.66	< 0.001

**Figure 4:**
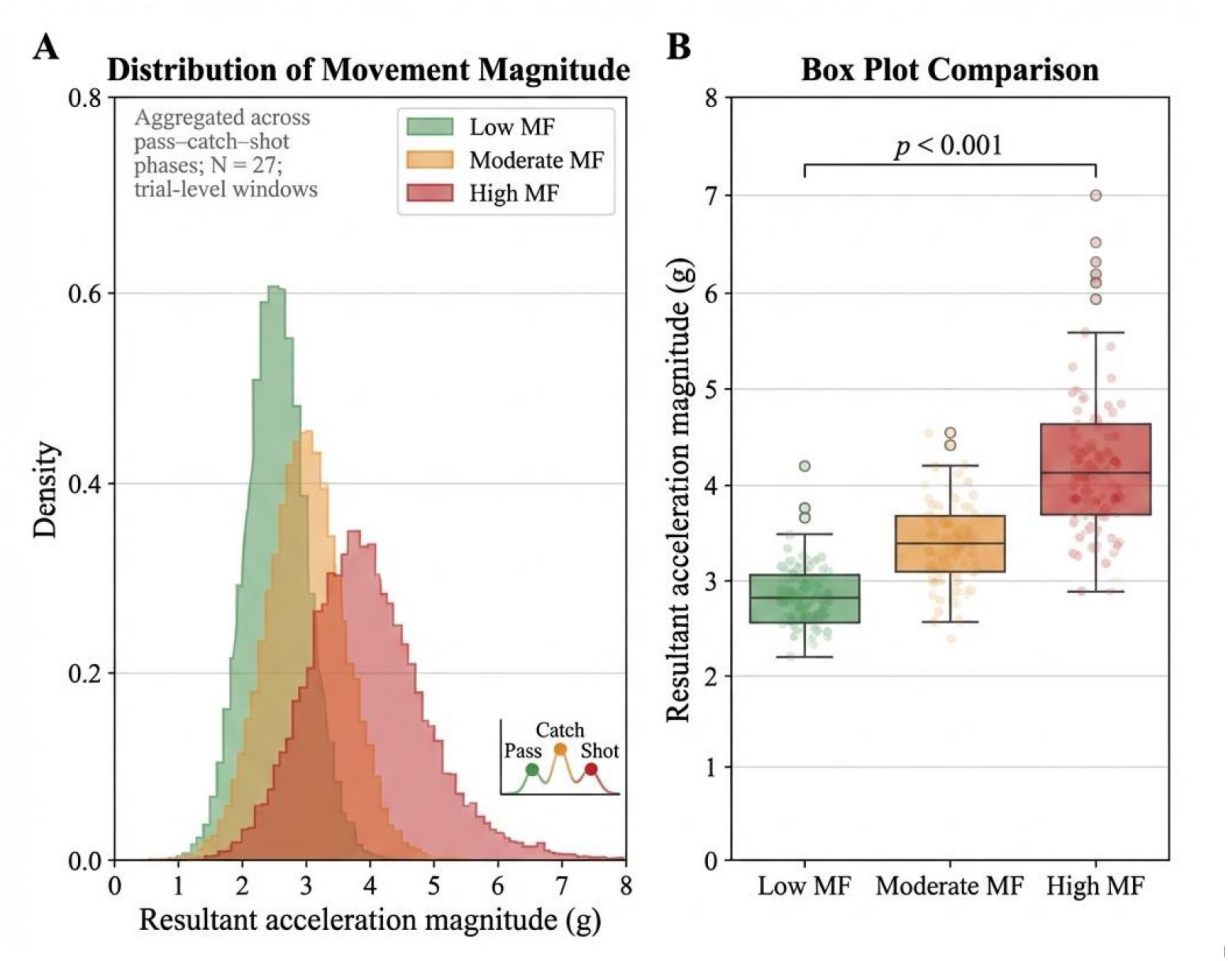
Fatigue-dependent variations in movement kinematics during the pass-catch-shot sequence. **(A)** Overlaid density-normalized histograms illustrating the broadened distribution of resultant acceleration magnitude as mental fatigue increases. **(B)** Boxplots comparing resultant acceleration magnitude across the three fatigue conditions, with individual data points and outliers depicted to summarize inter-condition dispersion.

The changes in movement pattern distribution are illustrated in Figure 4. Figure 4A shows that the distribution of resultant acceleration magnitude became progressively broader and less concentrated as fatigue increased, indicating greater variability in movement output across the pass–catch–shot sequence. Figure 4B further confirms this pattern, with higher acceleration magnitude and wider dispersion under moderate and high fatigue conditions, reflecting reduced movement stability and less consistent motor control.

### 3.4 Psychophysiological correlates of performance decline

As detailed in Table 5, mental fatigue elicited clear psychophysiological alterations across the experimental conditions. To rigorously quantify the relationships between these physiological shifts and behavioral impairments, within-subject condition-aggregated data were utilized for all correlation analyses. This approach ensured that the derived associations accurately reflected intra-individual cross-domain coupling while respecting the repeated-measures structure of the dataset. Generally, a greater psychophysiological burden correlated with diminished decision accuracy, reduced sequence success rates, and degraded movement stability.

**Table 5:** Psychophysiological markers across conditions and their associations with performance.

Psychophysiological marker	Low MF	Moderate MF	High MF	Decision accurac*y* (%) *r* (*P*)	Sequence success rate (%) *r* (*P*)	Composite consistenc*y* score *r* (*P*)
Mean HR during trials (bpm)	118.6 ± 10.8	123.9 ± 11.5	129.7 ± 12.4	−0.44 (0.021)	−0.41 (0.033)	−0.39 (0.046)
RMSSD (ms)	39.8 ± 10.6	34.7 ± 9.4	28.9 ± 8.8	0.48 (0.011)	0.46 (0.015)	0.43 (0.025)
SDNN (ms)	56.2 ± 13.1	51.1 ± 12.0	44.8 ± 11.3	0.39 (0.045)	0.37 (0.056)	0.36 (0.064)
LF/HF ratio (a.u.)	1.61 ± 0.72	1.94 ± 0.83	2.43 ± 0.98	−0.32 (0.10)	−0.35 (0.073)	−0.38 (0.049)
EDA tonic level, SCL (μS)	5.26 ± 1.54	5.88 ± 1.63	6.63 ± 1.78	−0.36 (0.062)	−0.40 (0.039)	−0.34 (0.079)
EDA phasic activity, SCR rate (min^−1^)	6.2 ± 2.1	7.4 ± 2.4	8.9 ± 2.8	−0.42 (0.028)	−0.45 (0.019)	−0.41 (0.034)
Recovery slope (HR, bpm/min)†	−4.8 ± 1.7	−3.9 ± 1.6	−3.1 ± 1.5	−0.40 (0.039)	−0.43 (0.024)	−0.38 (0.050)
RPE during trials (0–10)	3.1 ± 1.2	4.0 ± 1.3	5.2 ± 1.4	−0.47 (0.013)	−0.49 (0.009)	−0.45 (0.018)
Perceived mental effort (0–10)	3.6 ± 1.3	4.8 ± 1.4	6.1 ± 1.5	−0.51 (0.006)	−0.48 (0.010)	−0.46 (0.014)

**Figure 5:**
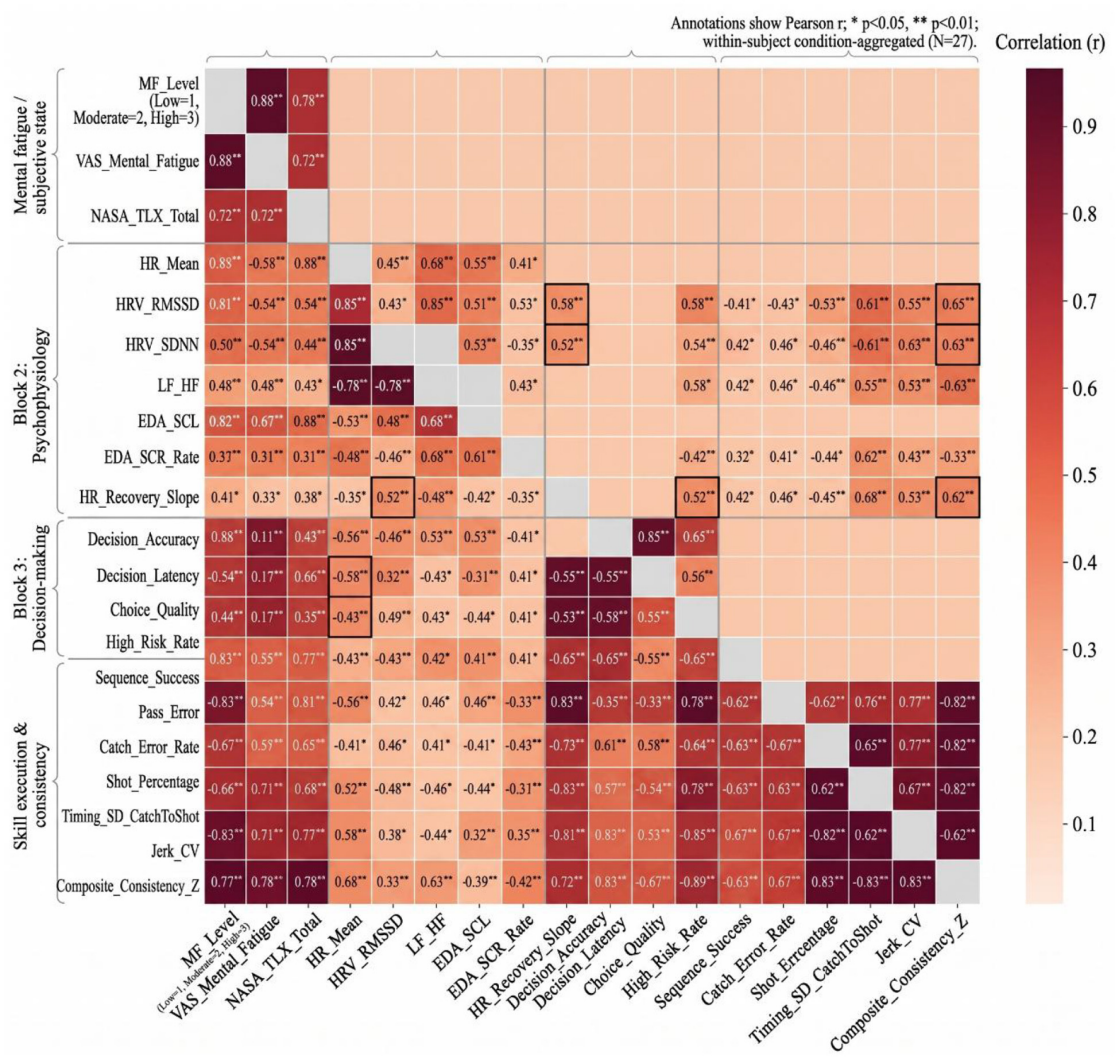
Comprehensive cross-domain correlation matrix illustrating the coupling among psychophysiological markers, decision-making metrics, and skill-consistency indices. To accurately capture generalized functional relationships rather than transient trial-level noise, Pearson correlation coefficients were computed using participant-level summaries aggregated across all three experimental conditions (N = 27).

This comprehensive cross-domain pattern is further mapped in Figure 5. Although the resulting correlation matrix is visually dense, presenting the complete array of pairwise associations is essential to demonstrate the integrated nature of fatigue. It visually confirms that psychophysiological markers did not fluctuate as isolated physiological responses, but rather formed a highly interconnected network with specific tactical decisions and kinematic stability indices.

**Figure 6:**
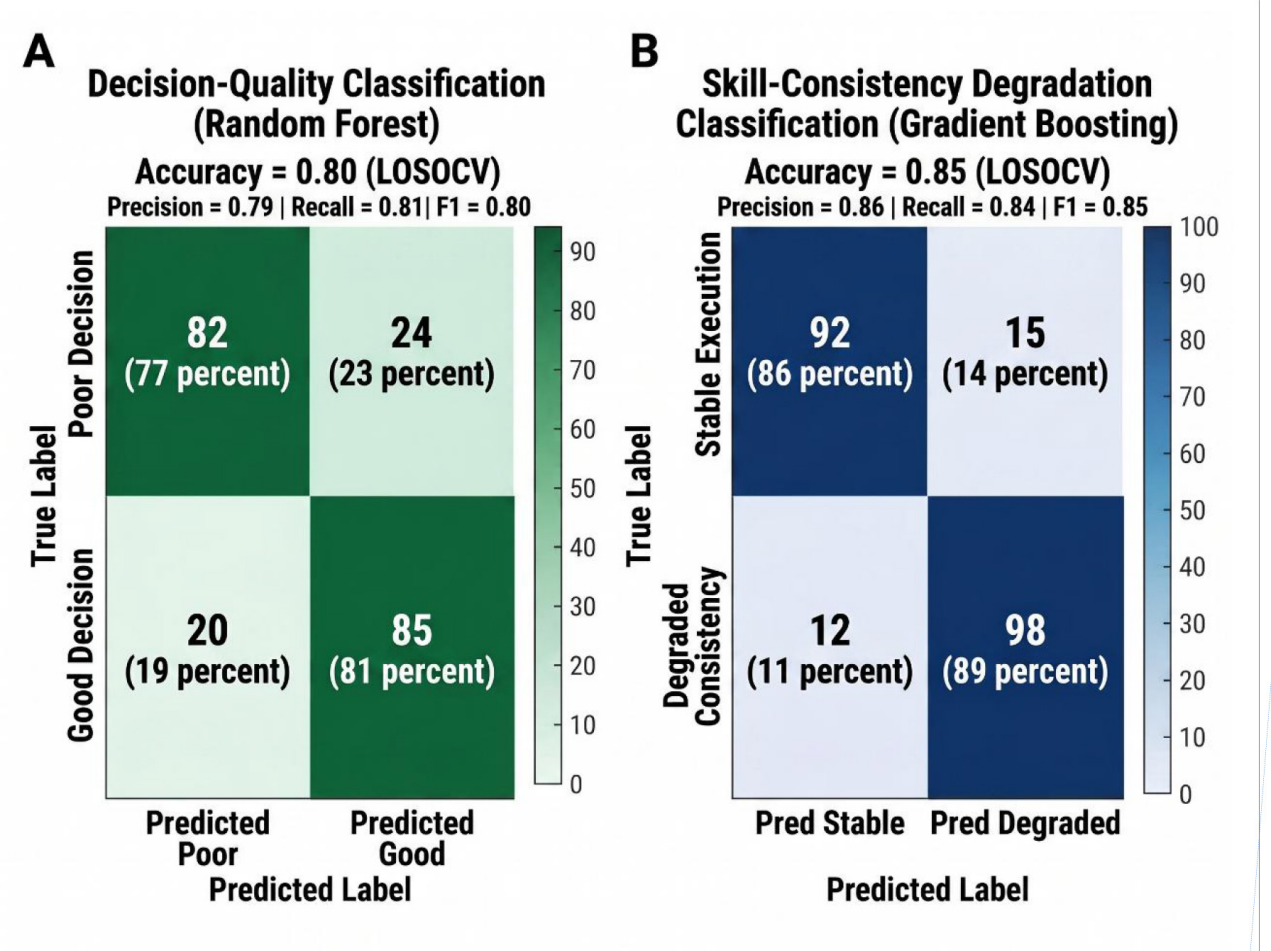
Machine learning classification matrices for fatigue-induced performance states. **(A)** Confusion matrix for decision-quality classification utilizing a Random Forest model. **(B)** Confusion matrix for skill-consistency degradation classification utilizing a Gradient Boosting model. Both algorithms were trained on standardized cross-modal features and evaluated through a strict Leave-One-Subject-Out Cross-Validation approach to guarantee predictive generalizability across participants.

Figure 6 provides supplementary model-based evidence for state discrimination, utilizing the robust Leave-One-Subject-Out Cross-Validation (LOSOCV) strategy detailed in the methodology. Specifically, Figure 6A demonstrates that the Random Forest algorithm successfully classified fatigue-related decision-quality states using the fused psychophysiological feature set. Similarly, Figure 6B illustrates comparable discriminative capacity for skill-consistency degradation states via the Gradient Boosting framework. Although these classification results do not replace the primary inferential findings, they further support the robustness of the observed psychophysiological-performance coupling and highlight the potential utility of these markers for identifying fatigue-related performance risk.

The multivariable results presented in Table 6 confirm that mental fatigue exerts an independent influence on basketball performance even after adjusting for psychophysiological dynamics. Following the purposeful selection strategy detailed in our statistical analysis, only significant bivariate correlates were retained as predictors. This approach ensured that the final models remained statistically parsimonious while accurately capturing the independent contributions of autonomic regulation and cognitive load to performance impairment.

**Table 6:** Multivariable mixed-effects models: the independent effects of mental fatigue and psychophysiological dynamics on outcomes.

Outcome (dependent variable)	Predictor (fixed effect)	β (SE)	95% CI	*P* value
Decision accuracy (%)	Moderate MF (vs. Low)	−3.4 (1.6)	−6.6 to −0.2	0.036
	High MF (vs. Low)	−7.9 (1.8)	−11.5 to −4.3	< 0.001
	HRV RMSSD (z)	2.6 (1.0)	0.6 to 4.6	0.012
	EDA SCR rate (z)	−2.1 (0.9)	−3.9 to −0.3	0.023
	Trial block (per +1)	−1.0 (0.7)	−2.4 to 0.4	0.16
Decision latency (ms)	Moderate MF (vs. low)	41.8 (18.9)	4.0 to 79.6	0.031
	High MF (vs. low)	108.6 (22.7)	63.3 to 153.9	< 0.001
	HR mean (z)	29.7 (12.8)	4.1 to 55.3	0.024
	HR recovery slope (z)†	21.4 (10.9)	−0.3 to 43.1	0.054
	Trial block (per + 1)	14.3 (8.7)	−3.1 to 31.7	0.11
Sequence success rate (%)	Moderate MF (vs. low)	−4.9 (2.0)	−8.9 to −0.9	0.017
	High MF (vs. low)	−11.7 (2.4)	−16.5 to −6.9	< 0.001
	HRV RMSSD (*z*)	3.1 (1.1)	0.9 to 5.3	0.006
	EDA SCL (*z*)	−2.4 (1.0)	−4.4 to −0.4	0.018
	Trial block (per + 1)	−1.6 (0.8)	−3.2 to 0.0	0.051
Composite consistency (z)	Moderate MF (vs. *l*ow)	−0.24 (0.11)	−0.46 to −0.02	0.032
	High MF (vs. low)	−0.58 (0.13)	−0.84 to −0.32	< 0.001
	EDA SCR rate (z)	−0.18 (0.07)	−0.32 to −0.04	0.013
	HRV RMSSD (z)	0.16 (0.07)	0.02 to 0.30	0.028
	Trial block (per + 1)	−0.06 (0.05)	−0.16 to 0.04	0.24

## 4 Discussion

The results of this study demonstrate that the duration-based Stroop protocol successfully established a distinct gradient of mental fatigue, as evidenced by the systematic deterioration in subjective exhaustion and cognitive task efficiency (Quigley et al., 2024; Pham et al., 2025). While the within-subject randomized crossover design minimized the impact of inter-individual variability in technical proficiency and training history, the observed effects should be interpreted within the context of our specific cohort of trained male athletes (Wu et al., 2024). Beyond a simple reduction in success rates, these findings clarify the pathways through which cognitive exhaustion compromises basketball performance. The observed decline in decision accuracy and prolonged latency suggests that mental fatigue depletes the executive resources essential for rapid information processing and tactical response selection (Sonalcan et al., 2025; Zhu et al., 2026). In the high-pressure environment of basketball, even marginal delays in perceptual-cognitive judgment can trigger a cascading failure in the execution chain (Wang et al., 2024). Furthermore, our data extend previous research by showing that mental fatigue destabilizes the kinematic organization of motor sequences. The increased variability in phase timing and movement magnitude indicates that cognitive effort is required not only for decision-making but also for the continuous spatiotemporal regulation of motor output (Mortimer et al., 2024; Schampheleer and Roelands, 2024). When mentally fatigued, athletes appear less capable of compensating for minor perturbations during the pass–receive–shot sequence, leading to compounded mechanical inefficiencies (Lismane et al., 2026).

Crucially, the independent predictive value of autonomic markers—specifically heart rate variability and electrodermal activity—substantiates the cross-domain nature of this impairment (Fortes et al., 2025). The dynamic shift toward sympathetic dominance under high cognitive load serves as a physiological precursor to behavioral decline. Although the present laboratory setting limits direct ecological generalization, these integrated psychophysiological dynamics provide a robust framework for understanding performance risk in cognitively demanding sports (Qian et al., 2025).

## 5 Conclusion

In conclusion, this study demonstrates that mental fatigue exerts a systematic negative impact on basketball performance by disrupting the integration of tactical judgment and motor execution. As cognitive exhaustion intensified, participants exhibited a coordinated decline in decision accuracy and kinematic consistency, leading to a marked reduction in overall sequence success. These behavioral impairments were closely coupled with autonomic dysregulation, specifically reflected by elevated sympathetic arousal and reduced parasympathetic control. These findings suggest that psychophysiological markers may serve as sensitive indicators for monitoring cognitive load in basketball. Integrating objective autonomic assessments into training regimens could provide a more comprehensive view of an athlete's functional state. Future investigations should validate these integrated dynamics in competitive game environments to confirm the longitudinal utility of psychophysiological monitoring for performance management.

## Data Availability

The raw data supporting the conclusions of this article will be made available by the authors, without undue reservation.
